# Impact of preoperative risk factors on outcome after gastrectomy

**DOI:** 10.1186/s12957-020-1790-6

**Published:** 2020-01-24

**Authors:** Ann-Kathrin Eichelmann, Meltem Saidi, Kirsten Lindner, Christina Lenschow, Daniel Palmes, Andreas Pascher, Richard Hummel

**Affiliations:** 1 Department of General, Visceral and Transplant Surgery, University Hospital Münster, Albert-Schweitzer-Campus 1, W1, 48149 Münster, Germany; 2 Department of Surgery, University Hospital of Schleswig-Holstein, Ratzeburger Allee 160, 23538 Lübeck, Germany; 3 Department of General, Visceral, Vascular and Pediatric Surgery, University Hospital of Würzburg, Oberdürrbacher Straße 6, 97080 Würzburg, Germany

**Keywords:** Gastric cancer, Gastrectomy, Risk factors, P-/O-POSSUM, National Surgical Quality Improvement Program Surgical Risk Calculator

## Abstract

**Background:**

Gastrectomy is associated with relevant postoperative morbidity. However, outcome of surgery can be improved by careful selection of patients. The objective of the current study was therefore to identify preoperative risk factors that might impact on patients’ further outcome after surgical resection.

**Methods:**

Preoperative risk factors having respectively different surgical risk scores for major complex surgery (including Cologne Risk Score, p-/o-POSSUM, and NSQIP risk score) of patients that underwent gastrectomy for AEG II/III tumors and gastric cancer were correlated with complications according to Clavien-Dindo and outcome. Patients who underwent surgery in palliative intention were excluded from further analysis.

**Results:**

Subtotal gastrectomy was performed in 23%, gastrectomy in 59%, and extended gastrectomy in 18% in a total of 139 patients (mean age: 64 years old). Thirty six percent experienced a minor complication (Dindo I-II) and 24% a major complication (Dindo III-V), which resulted in a prolonged hospital stay (*p* < 0.001). In-hospital mortality (=Dindo V) was 2.5%. Besides age, type of surgical procedure impacted on complications with extended gastrectomy showing the highest risk (*p* = 0.005). The o-POSSUM score failed to predict mortality accurately. We observed a highly positive correlation between predicted morbidity respectively mortality and occurrence of complications estimated by p-POSSUM (*p* = 0.005), Cologne Risk (*p* = 0.007), and NSQIP scores (*p* < 0.001).

**Conclusion:**

The results demonstrate a significant association between different risk scores and occurrence of complications following gastrectomy. The p-POSSUM, Cologne Risk, and NSQIP score exhibited superior performance than the o-POSSUM score. Therefore, these scores might allow identification and selection of high-risk patients and thus might be highly useful for clinical decision making.

## Introduction 

Gastric cancer represents a major public health issue as one of the most frequent cancers worldwide. The GLOBOCAN report of 2018, published by the World Health Organization (WHO), reported over 1 million new cases with 783,000 reported deaths worldwide, and gastric cancer was the third leading cause of death in 2018 [1]. The incidence varies depending on sex (male-to-female ratio about 2:1) and between different geographic regions, with highest rates being recorded in East Asia and Eastern Europe. Despite improvements in surgical and perioperative management, gastric cancer still remains difficult to cure, mainly because of the absence of early clinical symptoms. Advanced gastric cancers typically present a poor prognosis with reported overall 5-year survival rates of only about 25% for European countries [2].

Surgery is associated with complication rates ranging from 9–46% after total gastrectomy [3, 4], and occurrence of complications is known to adversely affect length of stay, readmission rates, quality of life, and costs [5, 6]. Most importantly, postoperative complications—in particular anastomotic leakage—impact on mortality, recurrence, and survival rates [7, 8]. Mechanisms by which postoperative complications affect patients’ prognosis are not yet fully understood, but one potential reason might be that a prolonged inflammatory response in the context of complications could enhance residual tumor cell growth [9]. Additionally, patients with severe complications are less likely to undergo adjuvant therapy, what may influence disease-free and overall survival as well [3]. This relationship has only recently been analyzed by a Dutch group in their nationwide study, severe postoperative complications—besides weight loss and poor health status—had a threefold increased likelihood of omission of adjuvant treatment. Especially surgical complications in contrast to non-surgical complications resulted in omission of adjuvant chemotherapy (OR 3.4 vs. 1.9) [10].

Therefore, stringent selection of patients for surgery might be a valuable tool for prevention of postoperative complications. This led to introduction of different classification systems for analysis of performance status, such as the “Karnofsky Index” or the “ASA (American Society of Anesthesiologists’ Physical Status Classification Scale) Classification” into daily clinical practice. However, the major disadvantage of these non-specific scores is that they do not provide adequate risk assessment for patients undergoing complex surgeries [11, 12]. Hence, more specific risk scores have been developed for complex surgeries such as esophagogastric surgery, including the POSSUM Score (Physiological and Operative Severity Score for the enumeration of Mortality and Morbidity) [13–15], the “Cologne Risk Score” [16], and the ACS NSQIP (American College of Surgeons National Surgical Quality Improvement Program) Surgical Risk Calculator [17]. All these scores assess a number of perioperative organ functions and treatment details and are a popular tool to predict surgical risk. However, there is so-far only limited data available on the impact of these risk scores for outcome prediction in gastric cancer patients.

The present study now aimed to thoroughly assess the impact of preoperative patient-related risk factors and different (general and specific) risk scores on outcome after surgery for gastric cancer or cancers that invade the gastroesophageal junction and mandate extended gastrectomy [Adenocarcinoma of the esophagogastric junction (AEG) II/III]. For this purpose, the individual patient’s risk was assessed via analysis of multiple aspects of patient’s fitness and general condition, organ functions, as well as via different preoperative risk scores including the “Karnofsky Index”, the “p-/o-POSSUM”, “Cologne Risk Score”, and NSQIP surgical risk. These data were compared with perioperative complications as per Clavien-Dindo classification.

## Methods

### Patient recruitment, preoperative workup, and staging

Between January 2006 and January 2017, all patients who underwent (extended/total/subtotal) gastrectomy for gastric cancer and AEG II/III tumors were enrolled into the study. Patients who underwent surgery with palliative intention were excluded from analysis of postoperative complications and survival. Furthermore, patients who underwent gastrectomy in combination with heated intraperitoneal chemotherapy (HIPEC) were excluded from the study.

Clinical investigations with regards to patient’s general condition and fitness included comprehensive medical history, physical examination, blood tests, ECG, X-ray of the chest, anesthesiological consultation, and additional examinations as indicated. Preoperative tumor staging included an upper endoscopy (with biopsy and ultrasound) and CT-scan (thorax/abdomen/pelvis). Upon completion of the diagnostics, all patients were discussed in an interdisciplinary tumor board, and treatment intent and potential further investigations (e.g., laparoscopy) or neoadjuvant therapy were discussed and initiated. When neoadjuvant therapy was initiated, restaging investigations were performed to assess clinical response to treatment in order to exclude progressive disease or development of metastasis under pretreatment.

### Standard surgical procedure and postoperative course

Depending on tumor localization and size, staging results, and histological examination (Laurén-classification), either extended, total, or subtotal gastrectomy was performed. In case of curative intent, en bloc D2-lymphadenectomy was performed. To restore intestinal continuity, an end-to-side esophagojejunostomy or gastrojejunostomy with Roux-en-Y reconstruction was performed using a circular stapler in most cases. Routinely, patients received an epidural catheter for optimal analgesia, a gastric tube as well as an abdominal drain. Postoperatively, patients were extubated immediately and transferred to intermediate care unit for at least 1 day. Oral nutrition was started at day one with 400 ml fluids and was increased after the third postoperative day according to clinical progress. In addition, patients received total parenteral nutrition until enteral nutrition was sufficient. If anastomotic leakage was suspected, endoscopy was performed immediately. Otherwise, there was no routine control of the anastomosis such as Gastrographin swallow performed.

### Study parameters

All patients had a detailed preoperative assessment of their demographics, general condition, specific organ functions, tumor characteristics, treatment procedures, and the postoperative course according to the parameters as described below:

#### Demographics, general conditions and specific organ functions

Characteristics including age, gender, and patients’ body mass index (BMI) were recorded. Potential body weight loss was categorized into weight loss < 10%, 10–20%, and > 20%. Alcohol intake was classified as “elevated alcohol consumption” when patients reported to drink more than one drink per day on a regular basis; in case of presence of alcohol-related organ damage, alcohol consumption was classified as “very elevated”. Nicotine abuse was classified as follows: < 5 cigarettes/day, 6–20 cigarettes/day, and > 21 cigarettes/day. Assessment of specific organ functions included coronary heart disease, chronic heart failure, drug-treated hypertension, peripheral vascular disease, liver cirrhosis, dialysis-dependent renal failure, and diabetes mellitus. Data collection based on a questionnaire, a detailed medical history, and respective medical specialist reports. Additionally, spirometry was used to assess pulmonary function.

### Preoperative general performance status

Preoperative general performance status was assessed using different classification systems

#### Clinical impression on first consultation

The surgeon described his own impression of the patient’s overall condition on the initial presentation in the outpatient clinic. For this purpose, the general condition was described as either “good”, “reduced”, or “poor”, depending on presentation of the patient during the interview and clinical examination.

#### Karnofsky performance status

The Karnofsky index is an instrument to assess quality of life respectively to quantify activities of daily life [18]. For this study, patients were classified into three groups: > 80% (normal activity with effort, some symptoms of disease), = 70% (cares for him/herself, unable to carry on normal activity), or < 60% (requires occasional assistance, but able to care for most of his/her personal needs).

#### Cologne Risk score

The “Preoperative risk analysis” published by Schroeder et al. enables estimation of the patient’s general condition by considering several organ functions including pulmonary, cardiac, renal, and hepatic functions [16]. The risk parameters are finally summarized in a total score allowing a categorization into three risk groups: “normal risk” (13–16 points), “moderately increased risk” (17–22 points), or “high risk” (23–38 points). Details of the comprehensive score are illustrated in Additional file 1.

#### P- and o-POSSUM scores

Both scores are based on the POSSUM score, which analyzes 12 physiological/clinical parameters (age, cardiac function, respiratory function, ECG, systolic blood pressure, pulse rate, hemoglobin levels, white blood count, urea levels, sodium levels, potassium levels, and Glasgow Coma Scale). Besides these parameters, the p-POSSUM score takes six operative parameters into account (operation type, multiple procedures, total blood loss, presence of malignancy, peritoneal seeding, and mode of surgery) and allows prediction of morbidity and mortality. The o-POSSUM score was derived to provide a dedicated scoring system for esophageal and gastric surgeries. In contrast to the p-POSSUM score, operative blood loss and number of procedures were excluded from multivariate analysis. While the p-POSSUM score predicts postoperative morbidity and mortality, the o-POSSUM scores predicts postoperative mortality only [15].

#### NSQIP risk score

Based on the 21 preoperative patient characteristics such as age, ASA, BMI, and comorbidities, the ACS NSQIP universal risk calculator estimates the chance of 15 unfavorable outcomes such as complication or death following surgery [17].

### Tumor characteristics

Based on the preoperative tumor staging as described above, characteristics of the tumor including histology (EAC/adenosquamous carcinoma), location (cardia, body, fundus, antrum/pylorus), Laurén classification, tumor size, tumor stage, nodal stage, and presence of metastasis were recorded. Postoperatively, tumor stage was assessed by the 7th UICC TNM staging. T, N, and M categories as well as the resection margins, the histological grading, vein and lymph node invasion, and number of involved and resected lymph nodes were recorded. When neoadjuvant therapy was administered, pathologic response was categorized in tumor regression grades according to Baldus.

### Treatment details

#### Neoadjuvant therapy and clinical response

Depending on preoperative tumor staging, neoadjuvant therapy was initiated in patients with locally advanced but potentially curable cancers if patients were deemed fit for pretreatment. Neoadjuvant treatment was initiated in form of chemotherapy in most cases. Chemotherapy was administered according to the ECF (epirubicin, cisplatin, 5FU), FLOT (oxaliplatin, docetaxel, leucovorin, 5FU), EOX (epirubicin, oxaliplatin, capecitabine), or PLF (cisplatin, leucovorin, 5FU) protocols. Only three patients (with AEG tumors) received neoadjuvant radiochemotherapy. Clinical response was assessed by restaging investigations: patients with a significant decrease of the tumor diameter measured on the CT scan respectively of the endoluminal tumor size as visualized by endoscopy were classified as “Clinical responders” or, otherwise, as “Clinical non-responders”. Parameters of surgical treatment included intention of surgery (palliative/curative), procedure (subtotal/extended/gastrectomy) technique of anastomosis (hand/stapler) and intraoperative blood loss.

### Postoperative course

Postoperatively, length of stay, occurrence of complications, in-hospital mortality, overall survival, and disease-free survival were recorded. Patients who underwent gastrectomy in palliative intention were excluded from analysis. Furthermore, patients who died during their stay in hospital (= in-hospital mortality) were excluded for calculation of long-term survival.

### Classification of surgical complications

The perioperative surgical complications were assessed based on the Clavien-Dindo classification and therefore defined as “any deviation from the normal postoperative course” [19]. In clinical everyday life, grades III and IV complications are of high importance because these complications require immediate surgical, endoscopic, or radiological interventions, or mandate treatment of single or multi-organ failure in intensive care units. Therefore, complications were classified as follows: no complications (grade 0), minor complications (grade I and II), and major complications (grade III-V).

### Statistical analysis

All data are presented as means with standard deviation unless otherwise stated. Statistical analysis was performed with SPSS 25.0 (SPSS, Chicago, USA) by using Chi-square tests for categorical variables, Pearson’s correlation for numeric variables, and one-way ANOVA / Kruskal–Wallis for numeric versus categorical variables. The Kaplan–Meier method with log-rank tests was used for assessment of overall and disease-free survival. A *p* value < 0.05 was considered to be statistically significant.

## Results

### Demographics and physical condition including risk classification

**Fig. 1:**
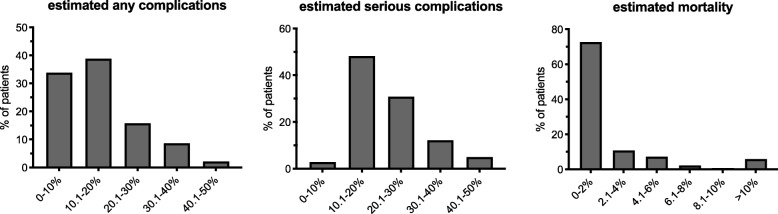
Estimated risk of any / serious complications respectively death estimated by the NSQIP risk score calculator

During the study period, a total of 139 patients with a mean age of 64 years old (24–91 years old) were enrolled into the study. Sixty nine percent of these patients were men. The majority of the study population (40%) was treated because of a carcinoma located in the corpus. Further details of the tumor characteristics are presented in Table 1. Preoperative physical condition, co-morbidities, respectively, other patient-related risk factors are presented in Table 2. The most frequent co-morbidities patients suffered from were cardiovascular diseases. In contrast, hepatic diseases for example were extremely rare. Only 11% of the study population did not suffer from any co-morbidity. Table 3 shows the patients’ distribution into the different risk groups according to Karnofsky and the Cologne Risk Score as well as the predicted morbidity/mortality rates calculated by p-/o-POSSUM while the surgical risk based on the NSQIP calculator is shown in Fig. 1.

**Table 1:** Demographics and clinic-pathologic characteristics (*n* = 139)

Characteristics	Number of patients	Percentage
Preoperative tumor findings		
Mean age	64 (24–91) years old	
Gender		
Male	96	69.1%
Female	43	30.9%
Localization		
Cardia	38	27.3%
Corpus	55	39.2%
Fundus	3	2.2%
Antrum/pylorus	43	30.9%
Histology		
Adenocarcinoma	138	99.3%
Adenosquamous carcinoma	1	0.7%
Laurén-classification		
Intestinal	54	38.8%
Diffuse	58	41.7%
Mixed	12	8.6%
Tumor stage		
uT1	16	11.5%
uT2	38	27.3%
uT3	72	51.8%
uT4	9	6.5%
Nodal stage		
uN positive	69	49.6%
uN negative	69	49.6%
Metastasis		
M0	126	90.6%
M1	11	7.9%
Mx	2	1.4%
Mean tumor size	3 (0.2–30) cm	
Neoadjuvant therapy		
Yes	57	41%
No	82	59%
Treatment		
Response to neoadjuvant therapy (*n* = 57)		
Clinical response (yes)	43	75.4%
Clinical response (no)	14	24.6%
Pathologic response (yes)	31	54%
Pathologic response (no)	26	45.6%
Intention		
Curative	120	86.3%
Palliative	19	13.7%
Timing		
Elective	138	99.3%
Emergency	1	0.7%
Procedure		
Subtotal	32	23%
Gastrectomy	82	59%
Extended	25	18%
Intraoperative blood loss		
≤ 100 ml	41	29.5%
101–500 ml	66	47.5%
501–1000 ml	29	20.9%
≥ 1001 ml	3	2.2%
Anastomosis		
Stapler	96	69.1%
Hand suture	43	30.9%
Postoperative tumor findings		
Tumor stage		
(y)pT0	6	4.3%
(y)pTis	1	0.7%
(y)pT1	26	18.7%
(y)pT2	30	21.6%
(y)pT3	47	33.8%
(y)pT4	29	20.9%
Nodal stage		
N0	62	44.6%
N1	35	25.2%
N2	16	11.5%
N3	26	18.7%
Metastasis		
M0	122	87.8%
M1	15	10.8%
Resection margin		
R0	122	87.8%
R1	15	10.8%
R2	2	1.4%
Histological grading		
G0	7	5%
G1	4	2.9%
G2	25	18%
G3	93	66.9%
Lymph nodes removed (mean)	23 (4–60)	
Lymph node invasion		
L0	87	62.6%
L1	45	32.4%
Invasion into vein		
V0	113	81.3%
V1	19	13.7%
Median hospital stay (range)	14 (1–120) days	
In-hospital mortality	4	2.9%
Survival rate	5.6 years	
Recurrence (*n* = 120)		
Yes	41	36.9%
No	69	62.2%
Recurrence-free survival	3.4 years

**Table 2:** Preoperative patient conditions and patient-related risk factors

Variables	Patients	Percentage
Coronary heart disease		
Yes	21	15.1%
No	118	84.9%
Heart insufficiency		
No	112	80.6%
Yes	27	19.4%
Drug-treated hypertension		
Yes	60	43.2%
No	79	56.8%
Arterial occlusive disease		
Yes	6	4.3%
No	133	95.7%
Vital capacity		
> 90%	108	77.7%
71–90%	22	15.8%
≤ 70%	9	6.5%
FEV1		
> 80%	113	81.3%
61–80%	15	10.8%
≤ 60%	11	7.9%
Liver cirrhosis		
None	136	97.8%
Child A	2	1.4%
Child B	1	0.7%
Child C	0	0
Creatinine Clearance		
> 95 ml/min	125	89.9%
≤ 95 ml/min	14	10.1%
Dialysis-dependent kidney insufficiency		
Yes	3	2.2%
No	136	97.8%
Insulin-dependent diabetes mellitus		
Yes	14	10.1%
No	125	89.9%
BMI		
20–25	68	48.9%
25.1–30	47	33.8%
> 30.1	24	17.3%
Weight loss		
< 10%	108	77.7%
10.1–20%	27	19.4%
> 20.1%	4	2.9%
Alcohol consumption		
Normal	125	89.9%
Elevated	12	8.6%
Very elevated	2	1.4%
Nicotine abuse/day		
< 5 cigarettes	103	74.1%
6–20 cigarettes	25	18%
> 21 cigarettes	11	7.9%

**Table 3:** Risk scores as potential predictors

Variables	Patients	Percentage
Patient’s general condition		
Good	96	69.1%
Reduced	37	26.6%
Poor	6	4.3%
Karnofsky index		
> 80	119	85.6%
70%	14	10.1%
< 60%	6	4.3%
Cologne Risk score		
Normal risk	31	22.3%
Moderate risk	69	49.6%
High risk	39	28.1%
POSSUM score (Morbidity)		
0–10%	1	0.7%
10.1–20%	25	18%
20.1–30%	24	17.3%
30.1–40%	23	16.5%
40.1–50%	8	5.8%
50.1–60%	20	14.4%
60.1–70%	15	10.8%
70.1–80%	6	4.3%
80.1–90%	9	6.5%
90.1–100%	8	5.8%
POSSUM score (Mortality)		
0–2%	71	51.1%
2.1–4%	22	15.8%
4.1–6%	19	13.7%
6.1–8%	5	3.6%
8.1–10%	3	2.2%
> 10%	19	13.7%
O-POSSUM score (Mortality)		
< 1%	133	95.7%
1–2%	6	4.3%

### Treatment, surgical details, and postoperative tumor characteristics

Fifty seven (41%) patients underwent neoadjuvant treatment [54 patients received neoadjuvant chemotherapy and 3 patients (with AEG II tumors) received neoadjuvant radiochemotherapy]. Following neoadjuvant treatment, 75% of the patients were classified as responders (= clinical response). Surgery was performed with curative intent in the majority of patients (86.3%). The main reason for palliative gastrectomy was bleeding. Subtotal gastrectomy was performed in 23%, gastrectomy in 59%, and extended gastrectomy in 18% of the patients using a stapler for anastomosis in 69%. For further details and postoperative tumor findings, see Table 1.

### Outcome: morbidity and mortality

Only patients that underwent surgery with curative intention were included for further analysis (*n* = 120). Forty percent of the patients did not suffer from any postoperative complication. See Table 4 for distribution of the patients according to Dindo. The median in-hospital stay was 14 days. In case of complications, hospital stay was prolonged (no complications: 12 days, I-II: 15 days, III-V: 32 days; *p* < 0.001).

**Table 4:** Postoperative complications according to Clavien-Dindo (patients who underwent surgery in palliative intention were excluded from analysis, *n* = 120)

	Patients (*n* = 120)	Percentage
Clavien-Dindo		
None	48	40%
I	23	19.2%
II	20	16.7%
III	20	16.7%
IV	6	5%
V	3	2.5%
Morbidity		
Yes	72	60%
No	48	40%

### Impact of demographics and tumor characteristics on morbidity and mortality

Age was identified as most important risk factor. The higher the patients’ age, the higher the chance of occurrence of complications (*p* = 0.001), overall morbidity (*p* = 0.005), mortality (*p* = 0.042) as well as with length of hospital stay (*p* = 0.008). Moreover, localization of the tumor as well as the uT-/M-stage impacted on complication and mortality rates: a significant increased risk was observed when the tumor was located in the cardia while the risk was lower for tumors located in the antrum (morbidity: *p* = 0.039, mortality: *p* = 0.006). A significant increased risk was also observed for patients suffering from higher uT-/M-stages (morbidity: uT-/M *p* = 0.022/0.001, mortality: uM-stage *p* < 0.001).

### Impact of treatment and postoperative tumor staging on morbidity and mortality

The type of surgical procedure impacted on the occurrence of complications with extended gastrectomy showing the highest risk of complications (*p* = 0.005) but not on mortality rates. Morbidity and mortality rates were independent from intraoperative blood loss, timepoint of surgery, or technique of anastomosis, though. Furthermore, complication and mortality rates were neither associated with administration of neoadjuvant (R)CT (morbidity: *p* = 0.082, mortality: *p* = 0.811), postoperative tumor staging, nor with clinical or pathologic response.

### Impact of patient-related risk factors and risk scores on morbidity and mortality

**Fig. 2:**
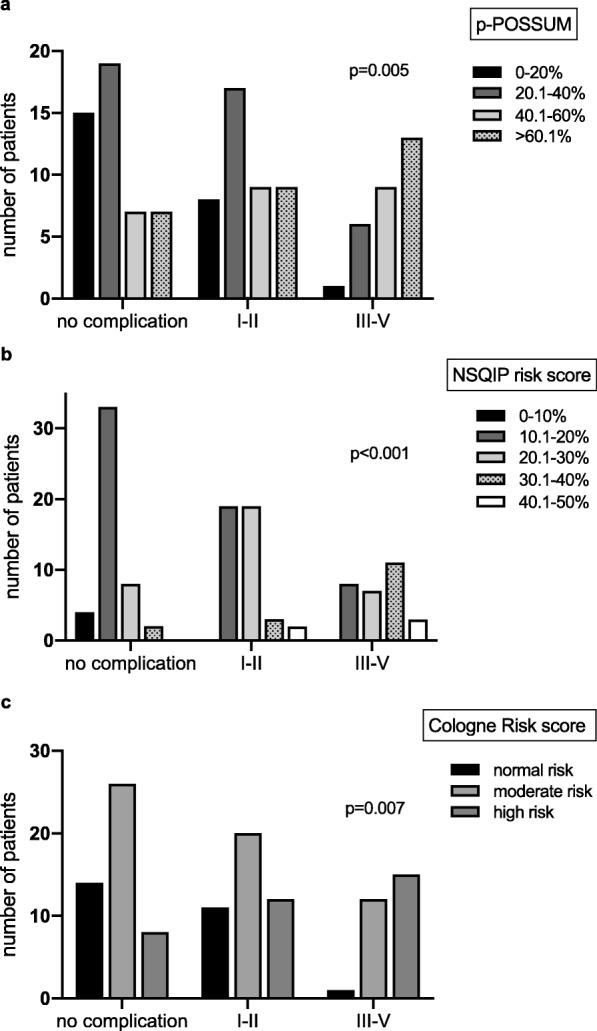
Correlation between preoperative risk as assessed according to p-POSSUM (**a**), NSQIP risk score (serious complications) (**b**) and Cologne risk score (**c**) and severity of postoperative complications as assessed via the Dindo classification

Neither the Karnofsky index nor the patient’s general condition correlated with occurrence of complications (*p* > 0.096). Regarding the scoring systems, mean morbidity rates were predicted as follows: p-POSSUM: 43%, NSQIP serious complications: 21%. The observed incidence of overall morbidity was 60%, respectively 21% for serious complications (Dindo III/IV) and therefore exactly as predicted by the NSQIP score. In correlation analysis, we observed a highly positive correlation between predicted morbidity and occurrence of complications estimated by the p-POSSUM (*p* = 0.005) and NSQIP score (*p* < 0.001, Fig. 2 a, b). In line with these findings, similar observations have been made for increasing preoperative risk according to the Cologne Risk score and increasing severity of postoperative complications (*p* = 0.007, Fig. 2c).

We did not observe a correlation between Karnofsky index, the patient’s general condition, nor the o-POSSUM score and mortality rates. The expected mortality rates predicted by the three other scoring systems were as follows: p-POSSUM: 4.4%, o-POSSUM: 0.36%, and NSQIP: 1.8%, while the observed incidence of mortality was 2.5%. Therefore, p-POSSUM overestimated mortality rates while the two other scores underestimated mortality, especially the o-POSSUM score. Correlation analysis showed a positive correlation between predicted mortality calculated by NSQIP score (*p* < 0.001) and p-POSSUM (*p* = 0.006) and death.

### Overall survival and disease-free survival

We observed a mean survival of 5.6 years. Survival rates correlated with localization (lowest overall survival when tumor was located in the cardia, *p* = 0.003), uN (*p* = 0.002), BMI (*p* = 0.024), weight loss (*p* = 0.001), smoking (*p* = 0.025), Karnofsky (*p* = 0.035), general status (*p* < 0.001), p-POSSUM morbidity and mortality (*p* = 0.019; *p* = 0.002), NSQIP scores (*p* < 0.004), type of surgery (*p* = 0.003), pT/N/L/V (*p* < 0.001), *R* (*p* = 0.12), and pathologic response (*p* = 0.002). Occurrence of complications shortened overall survival: e.g., patients suffered from a Dindo III/IV complication only showed an overall survival of 3.9 years (*p* = 0.05). Interestingly, patients who were attributed to the normal risk group according to the Cologne Risk score had an overall survival of 7.4 years while patients from the high-risk group only had survival rates of 3.6 years (*p* = 0.011).

Forty one patients (36.9%) suffered from recurrence; mean disease-free survival was 3.4 years (52–3782 days). Disease-free survival rates correlated with age (*p* = 0.049), localization of the tumor with shorter disease-free survival when located in the cardia (*p* < 0.001), uN (*p* = 0.002), length of tumor (*p* < 0.001), general status (*p* = 0.002), Karnofsky (*p* = 0.047), weight loss (*p* = 0.001), Cologne Risk score (*p* = 0.031), p-POSSUM morbidity (*p* < 0.001), NSQIP scores (*p* < 0.017), pT/N (*p* < 0.001), and pathologic response (*p* = 0.002).

## Discussion

Postoperative complications are known to influence outcome of patients undergoing gastrectomy, and despite improvements in surgical technique and perioperative management, surgery still bears relevant morbidity [3, 7]. High morbidity rates are also attributed to high numbers of elderly patients and patients in poor health that undergo extensive surgical procedures. Therefore, identification of patients with high risk for postoperative complications is of utmost importance in order to improve clinical decision-making with regards to personalized individual treatment planning. In this context, preoperative risk prediction using specific risk score such as the Cologne Risk score, the p-/o-POSSUM prediction models, or the NSQIP risk calculator might be of relevance. The current study aimed to thoroughly analyze the potential of general aspects such as demographics, tumor stage, physical condition, or co-morbidities as well as the potential of esophagogastric-specific preoperative risk scores to predict outcome after surgery for gastric cancer.

In contrast to unspecific performance status scores such as the Karnofsky index, which failed to predict outcome in our study population, we observed a significant correlation between the preoperatively estimated risk as calculated by the NSQIP score and morbidity and mortality. The estimated chance of serious complications for example was 21%, which was exactly in accordance with the observed incidence of serious complications. Mortality rates were slightly underestimated (estimated 1.8%, observed 2.5%). So far, the applicability of the NSQIP score, which was introduced in 2013, to patients undergoing gastrectomy has been assessed by only one other study, to the best of our knowledge [20]. In this multicenter study, Beal et al. included 965 patients who underwent resection of gastric adenocarcinoma and found variable results in terms of accuracy of the estimated risk. For example, highest correlation rates were observed for venous thromboembolism and lowest for renal failure. The authors conclude that the risk calculator represents a promising tool for risk prediction but needs further validation [20]. Despite limiting data regarding esophagogastric patients, the ACS NSQIP risk calculator was evaluated for a broad range of various study populations, ranging from head and neck cancer patients [21] to patients who underwent cystectomy [22]. Results of these studies were ambiguous: while the risk score demonstrated low accuracy in predicting postoperative outcomes in some cohorts [22, 23], it was considered as a reliable tool in the prediction of prognosis in other study populations [21, 24], suggesting that further studies are needed for validation.

Furthermore, we analyzed prediction models that were developed for esophagogastric surgery such as the Cologne Risk score. Latter was identified as accurate predictor of outcome in our study population. This score was first described by Schroeder et al. who demonstrated a correlation between the risk score and outcome of patients undergoing esophageal resection [16]. Our current results underline this hypothesis and are consistent with previous findings from our group for esophageal cancer surgery [25].

Also known as risk score for prediction of outcome following complex surgery is the POSSUM score, which was also considered in this study. Numerous authors investigated the potential of this well-known score in cancer patients within recent years, and some authors found an overestimation of risk by the factor two to three [26], especially in low-risk patients [27–29]. This observation led to the introduction of modifications of the POSSUM scoring system such as p-/o-POSSUM that consider operative parameters. Unfortunately, data on esophagogastric cancer patients are limited [30] and available data with regards to their potential to predict outcome are inconclusive. For example, Hong et al. identified p-/o-POSSUM as better predictors of postoperative mortality compared to POSSUM score [31]. In a review article that summarized 10 relevant publications, p-POSSUM showed the least overestimation compared to POSSUM and o-POSSUM scores and was therefore considered as most useful predictor of mortality [29]. In contrast, Bosch et al. showed that mortality after esophagectomy was best predicted by o-POSSUM, despite the fact that postoperative mortality was still overpredicted [32]. This observation is in line with findings from Gocmen et al. [33]. Other authors, however, resumed that the p-/o-POSSUM model is not a suitable tool to predict postoperative mortality following esophagogastric cancer resections accurately [34, 35].

In our study, o-POSSUM also failed to predict mortality, while p-POSSUM correlated with morbidity and mortality rates. However, the score underestimated morbidity (predicted: 43%, observed: 60%) and overestimated mortality (predicted: 4.4%, observed: 2.5%), as described by others [15]. However, it has to be considered that morbidity of 60% represents overall morbidity including Dindo I, which represent complications that do not require any particular therapy. Exclusion of Dindo I complications results in an observed morbidity rate of 40%, which is similar to the predicted morbidity estimated by p-POSSUM. It is also important to mention, though, that the p-POSSUM score also includes several operative parameters such as intraoperative blood loss. Hence, this score is in contrast to the NSQIP and Cologne Risk score and not a helpful tool for selection of patients preoperatively.

There are some limitations of the current study that need consideration. First, the current study is a retrospective cohort study on what causes a number of well-known limitations including for example the problem of incomplete or inconsistent acquisition of data. Second, the study is a single-center study with a limited number of patients. Summarized, a prospective study including a larger patient cohort is mandatory to confirm the current results, especially because of conflicting data regarding the potential of the different scores to predict outcome accurately as described above.

## Conclusion

The current study demonstrates a significant association between different risk scores and occurrence of complications following gastrectomy for gastric adenocarcinoma and AEG II/III tumors. Unspecific scores such as Karnofsky do not allow accurate prediction of outcome. In our study population, the Cologne Risk score and the NSQIP risk score exhibited superior performance than the o-POSSUM score. Our data clearly support the use of various parameters and scores for better patient selection and clinical decision making with the goal to reduce perioperative morbidity and mortality.

## Supplementary information

**Additional file 1.** Cologne Risk score.

## Data Availability

All data generated or analyzed during the study are included in this published article.

## Abbreviations

ACS NSQIP: American College of Surgeons National Surgical Quality Improvement Program
AEG: Adenocarcinoma of the esophagogastric junction
ASA: American Society of Anaesthesiologists’ Physical Status Classification Scale
BMI: Body mass index
EAC: Esophageal adenocarcinoma
ECG: Electrocardiogram
FEV1: Forced expiratory volume
HIPEC: Heated intraperitoneal chemotherapy
OR: Odds ratio
POSSUM: Physiological and Operative Severity Score for the enumeration of Mortality and Morbidity
WHO: World Health Organization
