# Supplementary material for: Impact of preoperative risk factors on outcome after gastrectomy
Source: World J Surg Oncol. 2020 Jan 24;18:17. doi: 10.1186/s12957-020-1790-6 (PMC6982377; doi:10.1186/s12957-020-1790-6)
Supplement: Supplementary file 1 — Additional file 1. Cologne Risk score. [file 12957_2020_1790_MOESM1_ESM.docx]

**Impact of preoperative risk factors on outcome after gastrectomy**

Corresponding author

Ann-Kathrin Eichelmann

Department of General, Visceral and Transplant Surgery

University Hospital Münster, Albert-Schweitzer-Campus 1, W1

48149 Münster, Germany

[Ann-Kathrin.Eichelmann@ukmuenster.de](mailto:Ann-Kathrin.Eichelmann@ukmuenster.de)

**Additional file 1** Cologne Risk Score [[17](#_ENREF_17)]

| **Variable** | **Classification of pathologic finding** | | |  |
| --- | --- | --- | --- | --- |
|  | **1 point**  **(normal risk)** | **2 points**  **(intermediate risk)** | **3 points**  **(high risk)** |  |
| **Pulmonary function**  VC  FEV_1_ | > 90%  >80% | 70-90%  60-80% | < 70%  < 60% | x 2 |
| **Cardiovascular function**  ECG  Myocardial infarction (date, number)  Blood pressure (BP) | Normal  No | CHD, PAD II-IV  Abnormal  > 6 months  > 145/95 mmHg | Heart failure NYHA III - IV  < 6 months, > 1  Systolic BP > 180 mmHg | x 3 |
| **Renal function**  Urea (mg/dl)  Creatinine (mg/dl)  Creatinine-clearance | < 50  < 1,1  95 - 160 | > 50  > 1,2  < 94 |  | x 1 |
| **Hepatic function** | Child A | Child B | Child C | x 2 |
| **Endocrine function** | Blood glucose level  55 - 115 mg% | Insulin-dependent diabetes mellitus |  | x 1 |
| **General status**  BMI (kg/m²)  Karnofsky index  Weight loss  **Alcohol consumption**  **Tobacco consumption** (cigarettes/d) | 20,0-24,9  > 80%  < 10%  Normal  none | 25-29,9 or <20,0  70 – 80%  10 – 20%  Elevated  5 -20 | > 30,0  < 70%  > 20%  High  > 20 | x 4 |
| **Neoadjuvant therapy** | yes |  |  | x 1 |

CHD = Coronary Heart Disease, PAD = Peripheral Arterial Disease
